# Protein intake during pregnancy and offspring body composition at 6 years: the Generation R Study

**DOI:** 10.1007/s00394-016-1255-4

**Published:** 2016-07-04

**Authors:** Myrte J. Tielemans, Eric A. P. Steegers, Trudy Voortman, Vincent W. V. Jaddoe, Fernando Rivadeneira, Oscar H. Franco, Jessica C. Kiefte-de Jong

**Affiliations:** 1 Department of Epidemiology, Erasmus MC, University Medical Center, Office Na-2907, PO Box 2040, 3000 CA Rotterdam, The Netherlands; 2 The Generation R Study Group, Erasmus MC, University Medical Center, Rotterdam, The Netherlands; 3 Department of Obstetrics and Gynecology, Erasmus MC, University Medical Center, Rotterdam, The Netherlands; 4 Department of Pediatrics, Erasmus MC, University Medical Center, Rotterdam, The Netherlands; 5 Department of Internal Medicine, Erasmus MC, University Medical Center, Rotterdam, The Netherlands; 6 Leiden University College, The Hague, The Netherlands

**Keywords:** Protein intake, Pregnancy, Body composition, Obesity, Offspring, Fetal programming

## Abstract

**Purpose:**

Intra-uterine exposure to protein may affect body composition and may increase the prevalence of childhood adiposity. Therefore, we examined whether protein intake during pregnancy is associated with offspring body composition at the age of 6 years and whether associations differ for animal protein and vegetable protein.

**Methods:**

We included 2694 Dutch mother–child pairs participating in a prospective population-based cohort in Rotterdam, the Netherlands. Energy-adjusted protein was measured in pregnancy using a food-frequency questionnaire and analyzed in quartiles. At a mean age of 6.1 ± 0.4 years, we measured children’s body mass index, and fat-free mass index and fat mass index using dual-energy X-ray absorptiometry. Outcomes were standardized for age and sex. BMI was used to classify children’s overweight status.

**Results:**

After adjustment for sociodemographic and lifestyle factors, a higher maternal protein intake was associated with a higher children’s fat-free mass index [difference 0.14 standard deviation (95 % CI 0.03, 0.25) for highest vs. lowest quartile of protein intake], but not with children’s fat mass index or body mass index. Comparable associations were found for animal protein and vegetable protein. Maternal protein intake was not associated with children’s overweight.

**Conclusions and relevance:**

This study suggests that higher protein intake during pregnancy is associated with a higher fat-free mass in children at the age of 6 years, but not with their fat mass. Our results do not suggest specific recommendations regarding maternal protein intake during pregnancy to prevent overweight in the offspring.

**Electronic supplementary material:**

The online version of this article (doi:10.1007/s00394-016-1255-4) contains supplementary material, which is available to authorized users.

## Introduction

The prevalence of childhood overweight is increasing worldwide [1]. Many overweight children will stay overweight or become obese when reaching adulthood [2], consequently increasing their risk of developing cardiovascular disease or type 2 diabetes later in life [3].

Childhood obesity and body mass index (BMI) can be influenced by several determinants such as genetic factors, children’s diet, and sedentary behavior [4–7]. In addition to this, intra-uterine exposures, such as pre-pregnancy BMI and maternal diet, have been suggested to affect body composition of the offspring via fetal programming [8, 9]. For example, higher maternal protein intake during pregnancy has been associated with body composition of the child; however, the results of several observational studies were inconsistent. Some studies have reported no associations [10, 11], whereas others found that higher maternal protein intake was associated with an increased risk of the offspring becoming overweight [12], or with a higher offspring lean mass [13]. The exact mechanisms through which maternal protein intake might influence children’s body composition have not been clarified, but may involve changes in the release of growth hormones or prenatal programming of child’s appetite [14, 15].

The effect of maternal protein intake on childhood body composition might differ depending on the source of protein. For instance, whether protein is animal-derived or vegetable-derived, because they differ in amino acid composition [16]. However, studies on the effects of different maternal protein sources on childhood body composition are scarce. We hypothesized that the association between maternal protein intake and offspring body composition would depend on the source of protein.

Therefore, the aim of our study was to assess whether maternal protein intake during pregnancy was associated with children’s body composition at the age of 6 years. Additionally, we assessed the differences in effect among protein sources (animal versus vegetable protein). Finally, we evaluated whether substitution of maternal protein for other macronutrients would influence these associations.

## Subjects and methods

This study was embedded in the Generation R Study, a prospective population-based birth cohort (Rotterdam, the Netherlands). Details of the study have been described in detail previously [17]. All women provided written informed consent at enrollment between April 2002 and January 2006. The study was approved by the Medical Ethics Committee of Erasmus Medical Center Rotterdam and conducted according to the World Medical Association Declaration of Helsinki.

### Study population

Out of 8976 women enrolled in the Generation R Study while pregnant, we restricted our analysis to women of Dutch ancestry (*n* = 4101). Ancestry was self-reported and defined according to the classification of Statistics Netherlands [18]. We excluded women with missing dietary information (*n* = 542), women with multiple pregnancies (*n* = 53) or no live childbirth (*n* = 24), and women who were lost-to-follow-up (*n* = 3). In our population of analysis, we included only mother–child pairs with available childhood body composition information at the age of 6 years (*n* = 2694; Supplemental Fig. 1).

### Maternal protein intake

Protein intake during pregnancy (i.e., total, animal, and vegetable protein) was assessed with a 293-item semiquantitative food-frequency questionnaire (FFQ) [19] that women received at enrollment at median 13.4 (IQR 12.2–15.5) weeks of gestation. The FFQ covered the average dietary intake of a Dutch diet over the previous three months. The average daily intake of energy, protein, and other nutrients was calculated using the Dutch food composition table 2006 [20]. Validation of the FFQ against three instances of 24-h dietary recall in 71 pregnant women of Dutch ancestry living in Rotterdam showed an intra-class correlation coefficient of 0.65 for energy-adjusted protein intake. There was no indication for systematic measurement error (Supplemental Fig. 2).

### Body composition measurements

Children visited the research center at a mean (±SD) age of 6.1 ± 0.4 years. We measured height (using a Harpenden stadiometer) and weight [using an electronic personal scale (Seca^®^)] to calculate their BMI (kg/m^2^). This BMI was also used to classify overweight status according to age- and sex-specific cutoffs [21].

During this visit, body composition was measured by dual-energy X-ray absorptiometry (DXA; iDXA; General Electrics-Lunar, 2008, Madison, WI, USA) following standardized procedures [22]. The DXA scanner calculated fat, lean, and bone mass of the total body and of specific body regions, using enCORE software (version 13; GE Healthcare). Fat-free mass index [FFMI (kg/m^2^); calculated as total fat-free mass divided by height squared] and fat mass index [FMI (kg/m^2^); total fat mass divided by height squared] were calculated. Additional outcome measurements were lean mass index (kg/m^2^, total fat-free mass minus total bone mass divided by height squared), total fat percentage (total body fat mass divided by total body mass times 100 %), and android/gynoid fat mass ratio (android fat mass divided by gynoid fat mass). All body composition outcomes were standardized for age and sex of the child and analyzed continuously.

### Covariates

At enrollment, we collected information by questionnaire on maternal age, educational level, folic acid supplement use, and parity. Additionally, weight and height were measured at the research center at enrollment to calculate BMI, and a fetal ultrasound was performed to establish gestational age. Energy, fat, and carbohydrate intake during pregnancy were measured using the FFQ described previously. Smoking and alcohol use were assessed during each trimester by questionnaire and categorized into never users, stopped when pregnancy was known, and continued use during pregnancy. Gestational weight gain (g/week) was calculated by subtracting maternal weight at enrollment from the weight in early third trimester and divided by the follow-up duration (weeks).

At birth, we collected information on gestational age at birth, birth weight *Z* score, sex, and hypertensive pregnancy complications (i.e., preeclampsia and pregnancy-induced hypertension) from delivery reports [23]. Preterm birth was defined as childbirth before 37 weeks of gestation. Breastfeeding practice at 2 months was assessed by a combination of delivery reports and questionnaires. Child protein and energy intake were measured using an FFQ at a median age of 12.9 (IQR 12.6–14.1) months in a subgroup of our population (*n* = 1591). At the age of 6 years, information on screen time (<2 vs. ≥2 h/day [24]) and participation in sports (yes/no) of the children was collected using a questionnaire.

### Statistical methods

Maternal protein intake was adjusted for total energy using the nutrient residual method to evaluate the effect of maternal protein intake independent of energy intake and to reduce the magnitude of measurement error [25]. We categorized protein intake into quartiles and used the lowest quartile (Q1) as the reference category. Because of skewed distributions, total body fat percentage and android/gynoid fat mass ratio were natural-log transformed.

We used multivariable linear regression models to assess the associations of maternal total, animal, and vegetable protein intake with childhood body composition measurements. Multivariable logistic regression models were used for childhood overweight.

The analyses were performed with energy-adjusted protein intake during pregnancy. Analyses with animal protein intake were adjusted for vegetable protein intake, and vice versa (*model 1*). Additionally, for the outcomes, total fat percentage and android/gynoid fat mass, *model 1* included also height of the child. The decision to include confounders in the multivariable regression models (*model 2*) was based on previous literature or a >10 % change in the effect estimate in *model 1*. The following confounders were considered: maternal age, educational level, parity, smoking and alcohol consumption in pregnancy, folic acid supplementation, maternal BMI at enrollment, energy intake, carbohydrate intake, gestational age at birth, breastfeeding, childhood sedentary time, and childhood physical activity. The confounders included are listed in the footnotes of the figures and tables. Potential intermediate factors were added to a separate multivariable model (*model 3*), namely gestational weight gain, hypertensive complications during pregnancy, and birth weight *Z* score. Effect modification was evaluated for gestational weight gain and child sex. In case of significant effect modification (*p* value for interaction term <0.05), stratified analyses were performed.

To evaluate whether the observed associations were due to a higher protein intake rather than a lower intake of another macronutrient, we assessed whether substituting protein with other macronutrients (e.g., carbohydrates and fat) had any effect on our results [26]. For example, the substitution model for replacing protein by carbohydrates included the macronutrients [in energy percent (*E*%)] protein, fat, and alcohol, but not the macronutrient carbohydrate. As a result, the regression coefficients for protein from these models reflect the effect of replacing 1 *E*% from carbohydrates with 1 *E*% of protein.

To evaluate the robustness of our findings, several secondary analyses were performed. First, we further adjusted our models for protein intake of the children (*n* = 1591). Second, we restricted analyses to women with a child born after 37 weeks of gestation, those without hypertensive complications in pregnancy, and to children with a normal birth weight (which we defined as a gestational age- and sex-adjusted birth weight between ±2 SD). Also, we excluded siblings (*n* = 185), and finally, we did not include the covariate child height in the multivariable *model 2* since height might also be associated with obesity [27].

To reduce bias due to missing data, missing covariates (0–17.7 %) were imputed using multiple imputation which includes fully conditioned specification of the imputation. Ten imputed datasets were created, and the analyses were performed in each dataset before the results were pooled by Rubin’s rules [28] taking into account uncertainty with the prediction of missing data. Details on the imputation procedure are described in Supplemental Table 1. All statistical analyses were performed in SPSS version 21.0 (IBM Corp., Armonk, NY, USA).

## Results

### Subject characteristics

Maternal and child characteristics are presented in Table 1. The main sources of protein in our study population were dairy products, meat and meat products, and nuts and seeds (together explaining 60 % of the variance in total protein intake). Mothers with a higher protein intake were, on average, older, had greater levels of education, more often non-smokers, and used more frequently folic acid supplementation than those with a lower protein intake (Supplemental Table 2).

**Table 1:** Baseline maternal characteristics, pregnancy outcomes, and children’s characteristics at the age of 6 years, the Generation R Study: Rotterdam, the Netherlands (*n* = 2694)

	Original data	Imputed data^a^
*Maternal characteristics (n* = *2694)*		
Gestational age at enrollment (weeks)	13.4 (12.2–15.5)	No missing values
Age (years)	31.7 ± 4.2	No missing values
Maternal education (%)		
Low and midlow (%)	11.9	12.0
Midhigh (%)	53.0	53.0
High (%)	35.1	35.1
Missing (%)	1.3	
Nulliparity (%)	61.9	61.8
Missing (%)	0.1	
Body mass index at enrollment (kg/m^2^)	23.4 (21.6–26.0)	23.4 (21.6–26.0)
Missing (%)	0.5	
Gestational weight gain^b^ (g/week)	503 ± 196	475 ± 204
Missing (%)	17.6	
Smoking during pregnancy		
Never (%)	75.9	76.1
Until pregnancy was known (%)	9.5	9.5
Continued (%)	14.6	14.4
Missing (%)	7.8	
Alcohol during pregnancy		
Never (%)	31.4	31.2
Until pregnancy was known (%)	16.7	16.7
Continued (%)	51.8	52.0
Missing (%)	8.5	
Alcohol consumption (g/day)	0.0 (0.0–0.7)	No missing values
Folic acid supplementation		
No (%)	9.2	9.5
Started <10 weeks of gestation (%)	90.8	90.5
Missing (%)	17.7	
Energy intake (kcal/day)	2153 ± 503	No missing values
Protein intake (g/day)		
Total protein	80 ± 19	No missing values
Animal protein	49 ± 14	No missing values
Vegetable protein	31 ± 9	No missing values
Protein intake (*E*%)		
Total protein	15 ± 2	No missing values
Animal protein	9 ± 2	No missing values
Vegetable protein	6 ± 1	No missing values
Pregnancy outcomes		
Hypertensive complications (%)	7.3	Not imputed
Missing (%)	3.2	Not imputed
Gender, boy (%)	50.1	No missing values
Birth weight (g)	3503 ± 541	3503 ± 540
Missing (%)	0.1	
Gestational age at birth (weeks)	40.0 ± 1.7	No missing values
Preterm birth (%)	4.2	No missing values
Breastfeeding at 2 months (%)	69.8	68.0
Missing (%)	15.0	
*Dietary intake of the children at 13 months of age*		
Energy intake (kcal/day)	1300 ± 342	Not imputed
Missing (%)	40.9	
Protein intake (g/day)	41 ± 11	Not imputed
Protein intake (*E*%)	13 ± 2	Not imputed
Missing (%)	40.9	
*Children’s characteristics at 6* *years of age*		
Age (years)	6.1 ± 0.4	No missing values
Playing sports (%)	50.0	49.9
Missing (%)	6.3	
≥2 h/day screen time (%)	19.9	20.8
Missing (%)	15.2	
Height of the children (cm)	120 ± 6	No missing values
Overweight/obese (%)	11.3	Not Imputed
Missing (%)	0.2	
Body mass index (kg/m^2^)	15.7 (15.0–16.6)	No missing values
Fat mass index (kg/m^2^)	3.6 (3.1–4.2)	Not imputed
Missing (%)	2.6	
Fat-free mass index (kg/m^2^)	11.9 ± 0.8	Not imputed
Missing (%)	2.6	
Total fat percentage (%)	23 (21–27)	Not imputed
Missing (%)	2.6	
Android/gynoid fat mass ratio	0.24 (0.21–0.27)	Not imputed
Missing (%)	2.6	

Values represent % for categorical variables and for continuous variables mean ± SD or median (interquartile range)

^a^Percentages may not add up to 100 % because of pooling of the imputed datasets

^b^Weekly gestational weight gain (g/week) between enrollment around 13 weeks of pregnancy and early third trimester (around 30 weeks)

### Protein intake during pregnancy and body composition in childhood

Children of mothers in the highest quartile (Q4) of protein intake did not have a statistically significant higher BMI than children of mothers in the lowest quartile (Q1; Table 2). Both animal protein and vegetable protein intake were not associated with a higher childhood BMI in *model 1*, whereas higher animal protein as well as higher vegetable protein intake was associated with higher childhood BMI after adjustment for confounders (*model 2*, Table 2).

**Table 2:** Association of maternal protein intake during pregnancy with childhood body composition at the age of 6 years (*n* = 2694)

	Childhood body mass index (SDS, *n* = 2694)		Fat-free mass index (SDS, *n* = 2624)		Fat mass index (SDS, *n* = 2624)	
	Model 1^a^	Model 2^b^	Model 1^a^	Model 2^b^	Model 1^a^	Model 2^b^
	*β* (95 % CI)	*β* (95 % CI)	*β* (95 % CI)	*β* (95 % CI)	*β* (95 % CI)	*β* (95 % CI)
Total protein intake^c^						
Quartile 1	Reference	Reference	Reference	Reference	Reference	Reference
Quartile 2	−0.03 (−0.14, 0.07)	0.03 (−0.08, 0.14)	**0.10 (0.00, 0.20)**	0.10 (−0.00, 0.20)	−0.10 (−0.20, 0.00)	−0.02 (−0.12, 0.08)
Quartile 3	0.00 (−0.11, 0.11)	0.07 (−0.04, 0.18)	**0.12 (0.02, 0.22)**	0.10 (−0.00, 0.20)	−0.08 (−0.18, 0.02)	0.02 (−0.08, 0.13)
Quartile 4	0.02 (−0.08, 0.13)	0.09 (−0.03, 0.20)	**0.17 (0.07, 0.27)**	**0.14 (0.03, 0.25)**	−0.10 (−0.20, 0.00)	0.01 (−0.09, 0.12)
*p* for trend	0.56	0.10	**0.001**	**0.02**	0.09	0.61
Animal protein intake^c^						
Quartile 1	Reference	Reference	Reference	Reference	Reference	Reference
Quartile 2	0.03 (−0.07, 0.14)	0.08 (−0.03, 0.19)	0.10 (−0.00, 0.20)	0.10 (−0.00, 0.20)	0.01 (−0.09, 0.11)	0.07 (−0.03, 0.17)
Quartile 3	0.04 (−0.07, 0.14)	0.08 (−0.03, 0.19)	**0.11 (0.01, 0.21)**	0.09 (−0.01, 0.20)	−0.02 (−0.12, 0.08)	0.05 (−0.05, 0.15)
Quartile 4	0.07 (−0.04, 0.18)	**0.12 (0.00. 0.24)**	**0.18 (0.08, 0.28)**	**0.16 (0.05, 0.27)**	−0.03 (−0.14, 0.07)	0.05 (−0.06, 0.17)
*p* for trend	0.23	0.06	**0.001**	**0.01**	0.45	0.46
Vegetable protein intake^c^						
Quartile 1	Reference	Reference	Reference	Reference	Reference	Reference
Quartile 2	−0.07 (−0.18, 0.04)	0.03 (−0.07, 0.14)	0.06 (−0.04, 0.16)	0.07 (−0.03, 0.17)	−0.10 (−0.20, 0.01)	0.02 (−0.08, 0.12)
Quartile 3	−0.03 (−0.13, 0.08)	**0.11 (0.00, 0.22)**	**0.14 (0.04, 0.24)**	**0.15 (0.04, 0.25)**	−**0.11 (**−**0.21,** −**0.01)**	0.07 (−0.04, 0.17)
Quartile 4	−0.03 (−0.14, 0.08)	**0.13 (0.01, 0.24)**	**0.22 (0.12, 0.32)**	**0.22 (0.11, 0.33)**	−**0.19 (**−**0.30,** −**0.09)**	0.02 (−0.09, 0.13)
*p* for trend	0.75	**0.01**	**<0.001**	**<0.001**	**0.001**	0.58

Results from multivariable linear regression analyses, based on imputed data. The regression coefficients (95 % CI) reflect the difference in age- and sex-specific SDS of childhood body mass index, fat-free mass index, and fat mass index relative to the first quartile of energy-adjusted protein intake. Trend tests were conducted by using the quartiles of protein intake as a continuous variable in the model

Bold values indicate significant associations (*p*-value < 0.05)

*CI* confidence interval, *SDS* standard deviation score

^a^ *Model 1*: Vegetable and animal protein intake were additionally adjusted for each other

^b^ *Model 2*: *Model 1* further adjusted for maternal age, educational level, smoking and alcohol use and folic acid supplementation during pregnancy, maternal body mass index at enrollment, energy and carbohydrate intake during pregnancy, gestational age at birth, breastfeeding 2 months postpartum and, screen time of the children at 6 years of age

^c^Energy-adjusted protein intake using the nutritional residual method

Total maternal protein intake was not associated with childhood overweight at the age of 6 years [OR 1.13 (95 % CI 0.80, 1.59) Q4 vs. Q1], after adjusting for educational level, maternal alcohol, and folic acid supplementation during pregnancy (*p* value for trend = 0.31) and neither was the source of maternal protein intake associated with childhood overweight.

Higher total protein intake during pregnancy was associated with a higher FFMI in children aged 6 years in the unadjusted model (*model 1*) as well as in the multivariable adjusted model (*model 2*, Table 2). The effect estimates were comparable for animal and vegetable protein (Table 2). Maternal total protein intake remained significantly associated with childhood FFMI [difference 0.15 SD (95 % CI 0.04, 0.25) for Q4 vs. Q1, *p* value for trend = 0.01] after additional adjustment of the potential intermediate factors gestational weight gain, hypertensive complications, and birth weight (*model 3*). Total maternal protein intake was not associated with childhood FMI and neither was the intake of animal protein during pregnancy (Table 2). Vegetable protein intake was associated with lower FMI in *model 1*, but this association did not remain after adjustment for lifestyle factors and sociodemographic background (*model 2*, Table 2).

In Supplemental Table 3, we added the results from the main analyses that were performed in a non-imputed dataset. The magnitude of the effects was stronger in the imputed data, which was mainly seen in the analysis for vegetable protein intake. Nonetheless, the 95 % confidence intervals from the imputed and non-imputed data largely overlapped and were not statistically significantly different.

Total protein and vegetable protein intake, but not intake of animal protein, were associated with lower childhood total fat percentage in the unadjusted analysis (*model 1*), but after adjustment for confounders (*model 2*), these associations did not remain (Supplemental Table 4). Vegetable protein intake was associated with a lower android/gynoid fat mass ratio in the unadjusted model only, and no association was found with total protein or animal protein intake and android/gynoid fat mass ratio (Supplemental Table 4).

### Secondary analyses

We did not observe specific substitution effects when protein (*E*%) was exchanged for different types of macronutrients in the association with FFMI (Table 3). Additional adjustment for protein intake of the child at 14 months of age (*n* = 1558; 59 %) slightly attenuated the results with FFMI (Supplemental Table 5).

**Table 3:** Substitution of maternal protein intake with other macronutrients and its association with childhood fat-free mass index at the age of 6 years (*n* = 2624)

Fat-free mass index (SDS, *n* = 2624)	*β* (95 % CI)
Protein (*E*%)	
Substitution for carbohydrate	**0.03 (0.01, 0.04)**
Substitution for monosaccharides and disaccharides	**0.03 (0.01, 0.04)**
Substitution for polysaccharides	0.02 (−0.00, 0.04)
Substitution for fat	**0.03 (0.02, 0.05)**
Substitution for saturated fat	0.02 (−0.00, 0.05)
Substitution for unsaturated fat	**0.04 (0.02, 0.05)**
Substitution for alcohol	0.03 (−0.03, 0.10)
Animal protein (*E*%)	
Substitution for carbohydrate	**0.03 (0.01, 0.04)**
Substitution for monosaccharides and disaccharides	**0.02 (0.01, 0.04)**
Substitution for polysaccharides	**0.03 (0.01, 0.05)**
Substitution for fat	**0.03 (0.01, 0.05)**
Substitution for saturated fat	0.01 (−0.01, 0.04)
Substitution for unsaturated fat	**0.04 (0.02, 0.06)**
Substitution for alcohol	0.02 (−0.04, 0.09)
Vegetable protein (*E*%)	
Substitution for carbohydrate	**0.07 (0.04, 0.11)**
Substitution for monosaccharides and disaccharides	**0.10 (0.05, 0.15)**
Substitution for polysaccharides	**0.10 (0.04, 0.16)**
Substitution for fat	**0.08 (0.04, 0.12)**
Substitution for saturated fat	**0.09 (0.04, 0.14)**
Substitution for unsaturated fat	**0.11 (0.06, 0.17)**
Substitution for alcohol	0.07 (−0.00, 0.14)

The effect estimates can be interpreted as difference in fat-free mass index per exchange of 1 *E*% from protein or sources of protein with an isocaloric amount of another macronutrient, while keeping the other macronutrients constant. Analyses were adjusted for maternal age, educational level, smoking and alcohol use and folic acid supplementation during pregnancy, maternal body mass index at enrollment, gestational age at birth, breastfeeding 2 months postpartum, and screen time of the children at 6 years of age

Bold values indicate significant associations (*p*-value < 0.05)

*CI* confidence interval, *E%* energy percent, *SDS* standard deviation score

The association between maternal protein intake and childhood lean mass index was similar to those for FFMI (Supplemental Table 6). When we restricted the analyses to a healthy population (i.e., term birth, normal birth weight, and mothers without hypertensive complications), there were no large differences in effect estimates (Supplemental Table 7). Excluding siblings from our population or excluding current height of the children from the analyses did not change the effect estimates (*data not shown*).

## Discussion

The results of this observational study indicate that higher protein intake during pregnancy is associated with higher fat-free mass in the offspring at the age of 6 years, but not with fat mass. These associations were similar for animal and vegetable protein, and we did not observe any specific substitution effect of maternal protein for other macronutrients.

Our results suggest that the higher BMI in children of mothers with a higher animal or vegetable protein intake was driven by a higher fat-free mass in the offspring rather than a higher fat mass. This implies that BMI, a method frequently used to assess adiposity, is an inaccurate measurement of excess fat mass in children, a finding which has been addressed by Freedman et al. [29]. In addition, we found that maternal protein intake was not associated with childhood fat mass after taking into account differences in maternal lifestyle and sociodemographic factors, a finding in line with results from previous cohort studies [11, 13]. We also observed that adjustment for differences in lifestyle factors and sociodemographic variables changed some effect estimates considerably. This implies that the association between maternal protein intake during pregnancy and offspring body composition is complex and is influenced by socioeconomic and lifestyle factors.

Our finding that children of mothers with a higher protein intake had a higher fat-free mass could not be explained by maternal lifestyle and socioeconomic characteristics, nor could it be explained by gestational weight gain, hypertensive complications, birth weight, or by infant protein intake. Furthermore, the association was not different when excluding bone mass from the analyses (lean mass index). These results are in line with those of Brion et al. [13] who performed an observational study in 5534 mother–child pairs, which found that higher maternal protein intake was associated with higher lean mass, but not with fat mass, in the offspring at the age of 10 years. Furthermore, this study showed that maternal but not paternal protein intake was associated with children’s lean mass, suggesting intrauterine effects. Conversely, another smaller study (*n* = 264) reported no association of maternal protein intake with fat-free mass in the offspring at the age of 16 years [11].

We did not observe consistent differential effects for maternal animal or vegetable protein intake. A previous study that investigated the association between different sources of maternal protein intake and body composition in the offspring reported that animal, but not vegetable, protein intake during pregnancy was associated with higher BMI in the offspring [12]. However, this association was only found in female offspring [12]. Whether specific sources of maternal protein do in fact influence body composition differently requires further study. We did not observe any specific macronutrient substitution effect, which indicates that it does not matter whether maternal protein intake is increased at the expense of fat or carbohydrate.

Maternal protein intake might influence childhood body composition through several mechanisms. Protein intake is needed for the regulation and accretion of muscle mass, which is a major component of fat-free mass [30]. In line with our results, a study in pigs showed that a higher maternal protein intake during pregnancy led to a higher lean but not fat mass in the offspring [31]. Further analyses of skeletal muscle of the piglets revealed that the effect on muscle mass may be due to both increased myogenesis and muscular differentiation. Further potential mechanisms that could influence child growth may be changes in secretion of growth hormones [14] or prenatal programming of children’s appetite [15].

### Strengths and limitations

Strengths of this study are the prospective population-based design, the large sample size, the postnatal follow-up of the offspring through 6 years of age, and the collection of numerous confounding factors. A further strength is the detailed information we collected with regard to body composition measurements, since DXA has a high accuracy of measuring fat mass and other soft-tissue body composition components [32, 33].

However, some limitations should be considered when interpreting our results. A limitation of our study is the measurement of protein intake using an FFQ, which is not very precise. However, FFQs have been shown to be accurate in ranking participants according to their intake [34], and energy adjustment may have reduced the magnitude of measurement error [25]. Also, there might be measurement error of the anthropometric measurements (i.e. BMI during pregnancy) and other covariates. However, since these are measured before the objective outcome measurement (i.e., body composition of the child), this measurement error is most likely non-differential and not lead to differential associations between dietary protein intake during pregnancy and body composition of the child. Since we had no data on maternal physical activity during pregnancy, residual confounding due to maternal physical activity levels could influence our results. A third limitation is the restriction to women of Dutch ancestry in our analyses within this multi-ethnic prospective cohort study. While the inclusion of other ethnicities could have led to differential misclassification of dietary intake [35], the restriction with regard to ethnicity may reduce the external validity of our results. Also, the Generation R Study consisted of a higher percentage of women with higher socioeconomic status than those that were eligible to participate [17]. However, such a selection bias has not been found to influence exposure–outcome associations [36]. Finally, we did not have complete data on all included covariates; the percentage of missing covariates ranged between 0.0 and 17.7 %. We used multiple imputation procedure to impute these missing covariates since complete case analysis would result in a considerable loss of information and may lead to biased estimates [37].

## Conclusion

In conclusion, we found that higher protein intake during pregnancy is associated with higher childhood fat-free mass, but not with childhood fat mass. The associations did not differ for vegetable versus animal protein, and the associations were not explained by maternal lifestyle or sociodemographic factors. Also, it did not matter whether protein intake was substituted for maternal fat or carbohydrate intake. Our results do not implicate specific recommendations on maternal protein intake during pregnancy to prevent overweight in children; however, it may be relevant for discussions on the influence of healthy diet during pregnancy on offspring lean mass. Further research is needed to identify the underlying mechanisms related to the observed associations (i.e., potential pathways related to different amino acids).

## Electronic supplementary material

Below is the link to the electronic supplementary material.

Supplementary material 1 (DOCX 115 kb)
