# Supplementary material for: Protein intake during pregnancy and offspring body composition at 6 years: the Generation R Study
Source: Eur J Nutr. 2016 Jul 4;56(6):2151–60. doi: 10.1007/s00394-016-1255-4 (PMC5579175; doi:10.1007/s00394-016-1255-4)
Supplement: Supplementary file 1 — Supplementary material 1 (DOCX 115 kb) [file 394_2016_1255_MOESM1_ESM.docx]

**European Journal of Nutrition**

**ONLINE SUPPLEMENTARY MATERIAL**

**Protein intake during pregnancy and offspring body composition at 6 years: The Generation R Study**

Myrte J. Tielemans; Eric A.P. Steegers*; Trudy Voortman*; Vincent W.V. Jaddoe; Fernando Rivadeneira; Oscar H. Franco; Jessica C. Kiefte-de Jong

* Contributed equally

**Corresponding author**

Myrte Tielemans, Department of Epidemiology, Erasmus MC, University Medical Center, Office Na-2907, PO Box 2040, 3000 CA Rotterdam, the Netherlands. Phone: +31 (0) 10 7043351, Fax: +31 (0) 10 7044657. E-mail: m.tielemans@erasmusmc.nl

**Supplemental Fig 1.** Flow chart of the study population: the Generation R Study, Rotterdam, the Netherlands

Dutch women enrolled during pregnancy *n* = 4,101

*n* = 542 women excluded who did not receive FFQ, did not return FFQ or had implausible dietary data

Women with eligible FFQ data *n* = 3,559

*n* = 80 women excluded due to no live birth (n=24), multiple pregnancy (n=53) or loss to follow up (n=3)

Women with singleton live birth *n* = 3,479

*n* =785 mother-child pairs excluded due to missing body composition data at the age of 6 years

Eligible mother-child pairs for current study *n* = 2,694

Participation of the children in follow-up measurements at the age of 6 years

*n* = 2,694

Data available on:

- Anthropometrics: *n* = 2,694
- Body composition: *n* = 2,624

**Supplemental Fig 2.** Bland-Altman plot validation of protein intake comparing FFQ and 24-hour dietary recall method (n=71).


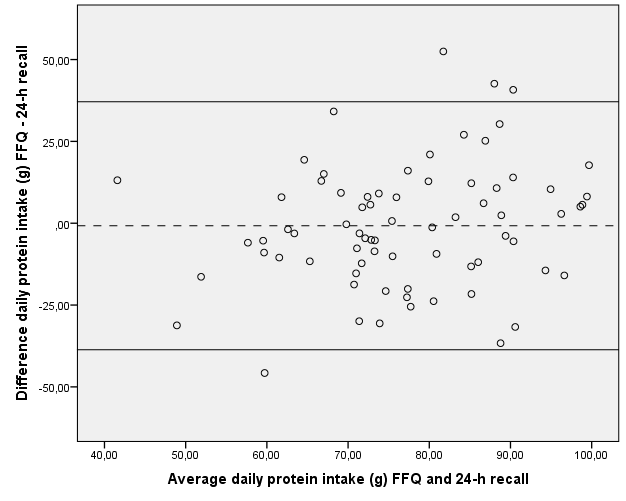


**Supplemental Table 1.** Details of the multiple imputation process^a^

|  | Multiple imputation [[1](#_ENREF_1),[2](#_ENREF_2)] |
| --- | --- |
| Software used | IBM SPSS statistics version 21 |
| Imputation method and key settings | Fully conditional specification (Markov chain Monte Carlo method); maximum iterations: 10 |
| No. of imputed data sets created | 10 |
| Variables included in the imputation procedure as both predictor variable as a variable to be imputed | Age mother at enrolment; Gravidity at enrolment; Parity at enrolment; Maternal educational level at enrolment; Pre-pregnancy maternal BMI; Maternal BMI at intake; Maternal alcohol consumption during pregnancy; Maternal smoking during pregnancy; Maternal folic acid use during pregnancy; Measured gestational weight gain; Gestational weight gain based on self-reported pre-pregnancy weight; Age partner at enrolment; BMI partner; Educational level partner at enrolment; Household income at enrolment; Gender child; Birth weight; Gestational age at birth; Standardized birth weight (Niklasson); Breastfeeding practice at the age of 2 months; Age of the children at follow-up; Height of the children at follow-up; Screen time of the children at the age of 6 years; Participation in sports of the children at the age of 6 years. |
| Variables added as predictors (not used in the main analyses) of missing data to increase plausibility of missing at random assumption | Maternal energy-adjusted protein intake during pregnancy; Maternal energy intake during pregnancy; Maternal serum folate level <18 weeks of gestation; Breastfeeding practice (ever versus never); Maternal educational level 6 years after enrolment; Household income 6 years after enrolment; Total energy intake of the children at the age of 14 months; Weight of the children at DXA measurement; Android/gynoid fat mass ratio of the children at follow-up (measured by DXA); Total fat percentage of the children at follow-up (measured by DXA); Fat-free mass index of the children at follow-up (measured by DXA); Fat mass index of the children at follow-up (measured by DXA); BMI-for-age of the children between 10-13 months; BMI-for-age of the children between 13-17 months; BMI-for-age of the children between 17-23 months; BMI-for-age of the children between 23-35 months; BMI-for-age of the children between 35-44 months; BMI-for-age of the children between 44-56 months; BMI of the children at follow-up; Overweight status of the children at follow-up. |
| Treatment of not normally distributed variables | Predictive mean matching |
| Treatment of binary/categorical variables | Logistic regression models |
| Population | For the multiple imputation we included those mothers with a Dutch ancestry, available dietary information, and those who visited the research center with their children at the age of 6 years (n=2,777) |

^a^ The missing values of the covariate ‘hypertensive complications during pregnancy’ were not imputed as a result of lacking predictive variables. **Abbreviations:** BMI: body mass index, DXA: dual-energy X-ray absorptiometry.

References

1. Sterne JA, White IR, Carlin JB, Spratt M, Royston P, Kenward MG, Wood AM, Carpenter JR (2009) Multiple imputation for missing data in epidemiological and clinical research: potential and pitfalls. BMJ 338:b2393

2. Rubin DB, Schenker N (1991) Multiple imputation in health-care databases: an overview and some applications. Stat Med 10 (4):585-598

**Supplemental Table 2.** Maternal and children’s characteristics presented per quartile of energy-adjusted maternal protein intake (n=2,694)^a^

|  |  | **Quartile 1**  n=673 | | **Quartile 2**  n=674 | **Quartile 3**  n=674 | **Quartile 4** n=673 | **P value** |
| --- | --- | --- | --- | --- | --- | --- | --- |
| Crude values of protein intake (g/day)^b^ |  | 65 (16,110) | | 74 (38,115) | 81 (49,120) | 95 (55,145) | **<0.001** |
| Energy-adjusted values of protein intake^b^ |  | -12 (-43,-8) | | -4 (-8,-0) | 3 (-0,7) | 13 (7,41) | **<0.001** |
| **Maternal Characteristics** |  |  | |  |  |  |  |
| Gestational age at enrolment (weeks) |  | 13.4 (12.2-15.8) | | 13.5 (12.4-15.7) | 13.4 (12.2-15.2) | 13.2 (12.2-15.4) |  |
| Age (years) |  | 30.8 ±4.5 | | 31.6 ± 4.3 | 32.0 ± 3.9 | 32.2 ± 4.0 | **<0.001** |
| Maternal education (%) Low and midlow (%) |  | 21.3 | | 10.5 | 8.4 | 7.5 | **<0.001** |
| Midhigh (%) |  | 55.2 | | 55.5 | 51.6 | 49.9 |  |
| High (%) |  | 23.5 | | 34.1 | 40.0 | 42.7 |  |
| *Missing (%)* |  | *1.5* | | *2.1* | *1.0* | *0.7* |  |
| Nulliparity (%) |  | 63.1 | | 59.7 | 63.1 | 61.6 | 0.53 |
| *Missing (%)* |  | *0.1* | | *0.3* | *0.0* | *0.1* |  |
| Body mass index at enrolment (kg/m^2^) |  | 23.4 (21.2-26.2) | | 23.3 (21.3-25.8) | 23.4 (21.9-25.8) | 23.7 (22.1-26.2) | 0.15 |
| *Missing (%)* |  | *0.6* | | *0.4* | *0.4* | *0.4* |  |
| Gestational weight gain^c^ (g/week) |  | 498 ±208 | | 509 ± 188 | 495 ± 193 | 509 ± 197 | 0.50 |
| *Missing (%)* |  | *18.1* | | *19.1* | *16.5* | *16.6* |  |
| Smoking during pregnancy Never (%) |  | 71.5 | | 75.8 | 79.2 | 77.2 | **0.004** |
| Until pregnancy was known (%) |  | 10.1 | | 9.0 | 8.5 | 10.5 |  |
| Continued (%) |  | 18.4 | | 15.3 | 12.3 | 12.4 |  |
| *Missing (%)* |  | *7.3* | | *10.5* | *5.8* | *7.6* |  |
| Alcohol during pregnancy Never (%) |  | 42.1 | | 29.9 | 26.9 | 26.8 | **<0.001** |
| Until pregnancy was known (%) |  | 16.8 | | 16.9 | 16.3 | 17.0 |  |
| Continued (%) |  | 41.1 | | 53.2 | 56.7 | 56.2 |  |
| *Missing (%)* |  | *8.2* | | *11.3* | *6.4* | *8.0* |  |
| Alcohol consumption (g/day) |  |  | |  |  |  |  |
| Folic acid supplementation No (%) |  | 11.7 | | 10.4 | 7.6 | 7.0 | **0.02** |
| Started < 10 wk of gestation (%) |  | 88.3 | | 89.6 | 92.4 | 93.0 |  |
| *Missing (%)* |  | *18.9* | | *20.3* | *16.3* | *15.3* |  |
| Energy intake (kcal/day) |  | 2,178 ±560 | | 2,104 ± 507 | 2,128 ± 472 | 2,200 ± 462 | **0.001** |
| Protein intake (g/day) Total protein |  | 66 ±16 | | 74 ± 15 | 82 ± 14 | 96 ± 16 | **<0.001** |
| Animal protein |  | 37 ±11 | | 44 ± 10 | 51 ± 9 | 63 ± 11 | **<0.001** |
| Vegetable protein |  | 30 ±9 | | 31 ± 9 | 31 ± 8 | 32 ± 9 | **<0.001** |
| Protein intake (E%) Total protein |  | 12 ±1 | | 14 ± 1 | 16 ± 1 | 18 ± 2 | **<0.001** |
| Animal protein |  | 7 ±1 | | 8 ± 1 | 10 ± 1 | 12 ± 2 | **<0.001** |
| Vegetable protein |  | 5 ±1 | | 6 ± 1 | 6 ± 1 | 6 ± 1 | **<0.001** |
| **Pregnancy outcomes** |  |  | |  |  |  |  |
| Hypertensive complications (%) |  | 8.3 | | 6.0 | 6.4 | 8.4 | 0.18 |
| *Missing (%)* |  | *3.9* | | *2.8* | *2.7* | *3.3* |  |
| Gender, boy (%) |  | 50.5 | | 51.6 | 47.9 | 50.4 | 0.58 |
| Birth weight (g) |  | 3,473 ±536 | | 3,500 ± 537 | 3,509 ± 553 | 3,530 ±536 | 0.27 |
| *Missing (%)* |  | *0.4* | | *0.0* | *0.0* | *0.0* |  |
| Gestational age at birth (weeks) |  | 39.9 ±1.7 | | 40.0 ± 1.8 | 40.0 ± 1.7 | 40.1 ±1.5 | 0.11 |
| Preterm birth (%) |  | 4.8 | | 4.6 | 4.6 | 2.8 | 0.24 |
| Breastfeeding at 2 months (%) |  | 60.7 | | 70.1 | 73.1 | 74.9 | **<0.001** |
| *Missing (%)* |  | *19.2* | | *13.8* | *13.9* | *13.1* |  |
| **Dietary intake of the children aged 13 months** | |  | |  |  |  |  |
| Energy intake (kcal/day) |  | 1,326 ± 365 | | 1,273 ± 335 | 1,310 ± 340 | 1,293 ± 326 | 0.15 |
| *Missing (%)* |  | *42.6* | | *38.3* | *40.9* | *41.9* |  |
| Protein intake (g/day) |  | 41 ± 12 | | 40 ± 11 | 42 ± 11 | 43 ± 11 | **0.001** |
| Protein intake (E%) |  | 12 ± 2 | | 13 ± 2 | 13 ± 2 | 14 ± 2 | **<0.001** |
| *Missing (%)* |  | *42.6* | | *38.3* | *40.9* | *41.9* |  |
| **Children’s characteristics aged 6 years** | | | **Quartile 1** n=673 | **Quartile 2**  n=674 | **Quartile 3**  n=674 | **Quartile 4** n=673 | **P value** |
| Age (years) |  | | 6.1 ±0.5 | 6.1 ± 0.4 | 6.1 ± 0.5 | 6.1 ± 0.4 | 0.39 |
| Playing sports (%) |  | | 48.1 | 48.1 | 50.6 | 52.9 | 0.26 |
| *Missing (%)* |  | | *6.7* | *8.2* | *5.3* | *5.1* |  |
| ≥ 2h/day screen time (%) |  | | 25.8 | 19.9 | 17.7 | 16.5 | **<0.001** |
| *Missing (%)* |  | | *16.3* | *18.0* | *13.1* | *13.4* |  |
| Height of the children (cm) |  | | 120 ±6 | 119 ± 6 | 119 ± 6 | 120 ± 6 | 0.41 |
| Overweight/obese (%) |  | | 13.0 | 9.7 | 11.1 | 11.5 | 0.30 |
| *Missing (%)* |  | | *0.3* | *0.1* | *0.0* | *0.3* |  |
| Body mass index (kg/m^2^) |  | | 15.6 (14.9-16.6) | 15.6 (14.9-16.6) | 15.7 (15.0-16.6) | 15.7 (15.0-16.6) | 0.75 |
| Fat mass index (kg/m^2^) |  | | 3.6 (3.2-4.3) | 3.5 (3.0-4.3) | 3.6 (3.2-4.3) | 3.5 (3.1-4.1) | 0.12 |
| *Missing (%)* |  | | *2.8* | *2.7* | *1.9* | *3.0* |  |
| Fat-free mass index (kg/m^2^) |  | | 11.8 ± 0.8 | 11.9 ± 0.8 | 11.9 ± 0.8 | 12.0 ± 0.8 | **0.03** |
| *Missing (%)* |  | | *2.8* | *2.7* | *1.9* | *3.0* |  |
| Total fat percentage (%) |  | | 23.7 (21.0-27.0) | 23.2 (20.0-26.6) | 23.5 (20.8-26.8) | 23.1 (20.3-26.2) | **0.02** |
| *Missing (%)* |  | | *2.8* | *2.7* | *1.9* | *3.0* |  |
| Android/gynoid fat mass ratio |  | | 0.24 (0.21-0.27) | 0.24 (0.20-0.27) | 0.24 (0.21-0.27) | 0.24 (0.21-0.27) | 0.21 |
| *Missing (%)* |  | | *2.8* | *2.7* | *1.9* | *3.0* |  |

^a.^ The values represent for continuous measures mean ± SD or median (interquartile range), and for dichotomous values the percentage of participants per category are presented. The missing values are also presented in percentages. The p-values were calculated using Analysis of Variance (ANOVA). ^b.^ Results for protein intake are presented as median and total range. ^c.^ Weekly gestational weight gain (g/week) between enrolment around 13 weeks of pregnancy and early-third trimester (around 30 weeks). **Abbreviations:** CI: confidence interval, E%: energy percent.

**Supplemental Table 3.** Association of maternal protein intake during pregnancy with childhood body composition at the age of 6 years, in non-imputed data

|  | **Childhood body mass index** (SDS) | | **Fat-free mass index** (SDS) | | **Fat mass index** (SDS) | |
| --- | --- | --- | --- | --- | --- | --- |
|  | ***Model 1*^1^** | ***Model 2*^2^** | ***Model 1*^1^** | ***Model 2*^2^** | ***Model 1*^1^** | ***Model 2*^2^** |
|  | n= 2,694 | n= 1,605 | n= 2,624 | n= 1,560 | n= 2,624 | n= 1,560 |
| **Total protein intake^3^** | β (95% CI) | β (95% CI) | β (95% CI) | β (95% CI) | β (95% CI) | β (95% CI) |
| Quartile 1 | *reference* | *reference* | *reference* | *reference* | *reference* | *reference* |
| Quartile 2 | -0.03 (-0.14, 0.07) | 0.00 (-0.14, 0.13) | **0.10 (0.00, 0.20)** | 0.10 (-0.00, 0.20) | -0.10 (-0.20, 0.00) | -0.05 (-0.17, 0.08) |
| Quartile 3 | -0.00 (-0.11, 0.11) | 0.05 (-0.09, 0.18) | **0.12 (0.02, 0.22)** | 0.10 (-0.00, 0.20) | -0.08 (-0.18, 0.02) | -0.02 (-0.14, 0.11) |
| Quartile 4 | 0.02 (-0.08, 0.13) | 0.04 (-0.10, 0.18) | **0.17 (0.07, 0.27)** | **0.14 (0.03, 0.25)** | -0.10 (-0.20, 0.00) | -0.04 (-0.17, 0.09) |
| *p for trend* | *0.56* | *0.47* | ***0.001*** | *0.10* | *0.09* | *0.61* |
| **Animal protein intake^3^** |  |  |  |  |  |  |
| Quartile 1 | *reference* | *reference* | *reference* | *reference* | *reference* | *reference* |
| Quartile 2 | 0.03 (-0.07, 0.14) | 0.05 (-0.08, 0.18) | 0.10 (-0.00, 0.20) | 0.10 (-0.00, 0.20) | 0.01 (-0.09, 0.11) | 0.07 (-0.03, 0.17) |
| Quartile 3 | 0.04 (-0.07, 0.14) | 0.04 (-0.10, 0.18) | **0.11 (0.01, 0.21)** | 0.09 (-0.01, 0.20) | -0.02 (-0.12, 0.08) | 0.05 (-0.05, 0.15) |
| Quartile 4 | 0.07 (-0.04, 0.18) | 0.09 (-0.05, 0.23) | **0.18 (0.08, 0.28)** | **0.16 (0.05, 0.27)** | -0.03 (-0.14, 0.07) | 0.05 (-0.06, 0.17) |
| *p for trend* | *0.23* | *0.25* | ***0.001*** | ***0.03*** | *0.45* | *0.92* |
| **Vegetable protein intake^3^** | |  |  |  |  |  |
| Quartile 1 | *reference* | *reference* | *reference* | *reference* | *reference* | *reference* |
| Quartile 2 | -0.07 (-0.18, 0.04) | -0.04 (-0.17, 0.10) | 0.06 (-0.04, 0.16) | -0.06 (-0.19, 0.08) | -0.10 (-0.20, 0.01) | 0.01 (-0.11, 0.14) |
| Quartile 3 | -0.03 (-0.13, 0.08) | 0.02 (-0.12, 0.15) | **0.14 (0.04, 0.24)** | 0.03 (-0.10, 0.17) | **-0.11 (-0.21, -0.01)** | 0.01 (-0.11, 0.14) |
| Quartile 4 | -0.03 (-0.14, 0.08) | 0.07 (-0.08, 0.22) | **0.22 (0.12, 0.32)** | 0.11 (-0.03, 0.25) | **-0.19 (-0.30, -0.09)** | 0.03 (-0.10, 0.16) |
| *p for trend* | *0.75* | *0.21* | ***<0.001*** | ***0.046*** | ***0.001*** | *0.66* |

Results from multivariable linear regression analyses in non-imputed data. The regression coefficients (95% CI) reflect the difference in age- and sex-specific SDS of childhood body mass index, fat-free mass index and fat mass index relative to the first quartile of energy-adjusted protein intake. Trend tests were conducted by using the quartiles of protein intake as a continuous variable in the model. ^1.^ *Model 1*: Vegetable and animal protein intake were additionally adjusted for each other. ^2.^ *Model 2*: *Model 1* further adjusted for maternal age, educational level, smoking and alcohol use and folic acid supplementation during pregnancy, maternal body mass index at enrolment, energy and carbohydrate intake during pregnancy, gestational age at birth, breastfeeding 2 months postpartum and screen time of the children at 6 years of age. ^3.^ Energy-adjusted protein intake using the nutritional residual method. **Abbreviations** CI: confidence interval, SDS: standard deviation score.

**Supplemental Table 4.** Association between protein intake during pregnancy and childhood total fat percentage and android/gynoid fat mass ratio at the age of 6 years

|  | **Total fat percentage** (SDS)  n=2,624 | | **Android/gynoid fat mass ratio** (SDS)  n=2,624 | |
| --- | --- | --- | --- | --- |
|  | ***Model 1*^1^** | ***Model 2*^2^** | ***Model 1*^1^** | ***Model 2*^2^** |
| **Total protein intake^3^** | β (95% CI) | β (95% CI) | β (95% CI) | β (95% CI) |
| Quartile 1 | *reference* | *reference* | *reference* | *reference* |
| Quartile 2 | **-0.13 (-0.22, -0.03)** | -0.06 (-0.15, 0.04) | -0.10 (-0.20, 0.01) | -0.03 (-0.14, 0.08) |
| Quartile 3 | -0.09 (-0.18, 0.01) | 0.00 (-0.09, 0.10) | -0.02 (-0.13, 0.09) | 0.06 (-0.06, 0.17) |
| Quartile 4 | **-0.15 (-0.24, -0.05)** | -0.05 (-0.15, 0.06) | -0.08 (-0.19, 0.03) | 0.00 (-0.12, 0.12) |
| *p for trend* | ***0.009*** | *0.67* | *0.36* | *0.60* |
| **Animal protein intake^3^** | |  |  |  |
| Quartile 1 | *reference* | *reference* | *reference* | *reference* |
| Quartile 2 | -0.01 (-0.10, 0.09) | 0.04 (-0.06, 0.13) | 0.06 (-0.05, 0.16) | 0.10 (-0.01, 0.21) |
| Quartile 3 | -0.04 (-0.13, 0.06) | 0.02 (-0.07, 0.12) | 0.04 (-0.07, 0.15) | 0.09 (-0.03, 0.20) |
| Quartile 4 | -0.08 (-0.18, 0.01) | -0.01 (-0.11, 0.10) | -0.01 (-0.12, 0.10) | 0.05 (-0.07, 0.17) |
| *p for trend* | *0.08* | *0.83* | *0.75* | *0.52* |
| **Vegetable protein intake^3^** | |  |  |  |
| Quartile 1 | *reference* | *reference* | *reference* | *reference* |
| Quartile 2 | **-0.12 (-0.22, -0.03)** | -0.02 (-0.12, 0.07) | -0.11 (-0.21, 0.00) | -0.02 (-0.13, 0.09) |
| Quartile 3 | **-0.12 (-0.22, -0.03)** | 0.03 (-0.07, 0.12) | **-0.12 (-0.23, -0.01)** | 0.01 (-0.11, 0.12) |
| Quartile 4 | **-0.23 (-0.32, -0.13)** | -0.40 (-0.14, 0.06) | **-0.18 (-0.29, -0.07)** | -0.03 (-0.15, 0.09) |
| *p for trend* | ***<0.001*** | *0.68* | ***0.003*** | *0.74* |

Results from multivariable linear regression analyses, based on imputed data. The regression coefficients (95% CI) reflect the difference in age- and sex-specific SDS of total fat percentage and android/gynoid fat mass ratio of the child relative to the first quartile of energy-adjusted protein intake. Trend tests were conducted by using the quartiles of protein intake as a continuous variable in the model. ^1.^ *Model 1:* Adjusted for height of the child aged 6 years. The vegetable and animal protein intake were additionally adjusted for each other. ^2.^ *Model 2*: *Model 1* additionally adjusted for maternal age, educational level, smoking and alcohol use and folic acid supplementation during pregnancy, maternal body mass index at enrolment, energy and carbohydrate intake during pregnancy, gestational age at birth, breastfeeding 2 months postpartum and screen time of the children at 6 years of age. ^3.^ Energy-adjusted protein intake using the nutritional residual method. **Abbreviations:** CI: confidence interval. SDS: standard deviation score.

**Supplemental Table 5.** Association between protein intake during pregnancy and childhood fat-free mass index at the age of 6 years, in the population with available protein intake of the children at 14 months of age (n=1,558)

|  | **Fat-free mass index** (SDS)  n=1,558 | | |
| --- | --- | --- | --- |
|  | ***Model 1*^1^** | ***Model 2*^2^** | ***Model 2**^3^** |
| **Total protein intake^4^** | β (95% CI) | β (95% CI) | β (95% CI) |
| Quartile 1 | *reference* | *reference* | *reference* |
| Quartile 2 | 0.04 (-0.09, 0.17) | 0.01 (-0.11, 0.14) | 0.01 (-0.12, 0.14) |
| Quartile 3 | 0.12 (-0.01, 0.24) | 0.09 (-0.04, 0.22) | 0.08 (-0.05, 0.21) |
| Quartile 4 | **0.14 (0.02, 0.27)** | 0.11 (-0.03, 0.25) | 0.09 (-0.05, 0.23) |
| *p for trend* | ***0.01*** | ***0.02*** | *0.12* |
| **Animal protein intake^4^** | |  |  |
| Quartile 1 | *reference* | *reference* | *reference* |
| Quartile 2 | 0.06 (-0.06, 0.19) | 0.05 (-0.07, 0.18) | 0.04 (-0.08, 0.17) |
| Quartile 3 | 0.09 (-0.03, 0.22) | 0.07 (-0.06, 0.21) | 0.06 (-0.08, 0.19) |
| Quartile 4 | **0.17 (0.04, 0.31)** | **0.16 (0.02, 0.30)** | 0.14 (-0.01, 0.28) |
| *p for trend* | ***0.009*** | ***0.03*** | *0.07* |
| **Vegetable protein intake^4^** | |  |  |
| Quartile 1 | *reference* | *reference* | *reference* |
| Quartile 2 | -0.05 (-0.18, 0.08) | -0.05 (-0.19, 0.08) | -0.06 (-0.19, 0.08) |
| Quartile 3 | 0.04 (-0.09, 0.17) | 0.04 (-0.10, 0.17) | 0.03 (-0.11, 0.17) |
| Quartile 4 | **0.17 (0.03, 0.31)** | **0.16 (0.01, 0.30)** | **0.15 (0.00, 0.30)** |
| *p for trend* | ***0.003*** | ***0.008*** | ***0.01*** |

Results from multivariable linear regression analyses, based on imputed data. The regression coefficients (95% CI) reflect the difference in age- and sex-specific SDS of the fat-free mass index of the child relative to the first quartile of energy-adjusted protein intake. Trend tests were conducted by using the quartiles of protein intake as a continuous variable in the model. ^1.^ *Model 1*: The vegetable and animal protein intake were additionally adjusted for each other. ^2.^ *Model 2*: *Model 1* additionally adjusted for maternal age, educational level, smoking and alcohol use during pregnancy, folic acid supplementation, maternal body mass index at enrolment, energy and carbohydrate intake during pregnancy, gestational age at birth, breastfeeding 2 months postpartum and screen time of the children at 6 years of age. ^3.^ *Model 2**: *Model 2* additionally adjusted for protein intake of the children at 14 months of age. ^4.^ Energy-adjusted protein intake using the nutritional residual method. **Abbreviations:** CI: confidence interval, SDS: standard deviation score.

**Supplemental Table 6.** Association between protein intake during pregnancy and childhood lean mass index at the age of 6 years (n= 2,624)

|  | **Lean mass index** (SDS)  n=2,624 | |
| --- | --- | --- |
|  | **Model 1**^1^ | **Model 2^2^** |
| **Total protein intake^3^** | β (95% CI) | β (95% CI) |
| Quartile 1 | *reference* | *reference* |
| Quartile 2 | **0.10 (0.00, 0.20)** | 0.09 (-0.01, 0.20) |
| Quartile 3 | **0.12 (0.02, 0.21)** | 0.10 (-0.01, 0.20) |
| Quartile 4 | **0.17 (0.07, 0.27)** | **0.14 (0.03, 0.25)** |
| *p for trend* | ***0.001*** | ***0.02*** |
| **Animal protein intake^3^** | |  |
| Quartile 1 | *reference* | *reference* |
| Quartile 2 | 0.09 (-0.01, 0.19) | 0.10 (-0.01, 0.20) |
| Quartile 3 | **0.10 (0.00, 0.20)** | 0.09 (-0.02, 0.19) |
| Quartile 4 | **0.18 (0.07, 0.28)** | **0.16 (0.04, 0.27)** |
| *p for trend* | ***0.001*** | ***0.01*** |
| **Vegetable protein intake^3^** | |  |
| Quartile 1 | *reference* | *reference* |
| Quartile 2 | 0.05 (-0.05, 0.15) | 0.07 (-0.03, 0.17) |
| Quartile 3 | **0.14 (0.04, 0.24)** | **0.15 (0.04, 0.25)** |
| Quartile 4 | **0.22 (0.12, 0.32)** | **0.22 (0.11, 0.33)** |
| *p for trend* | ***0.001*** | ***<0.001*** |

Results from multivariable linear regression analyses, based on imputed data. The regression coefficients (95% CI) reflect the difference in age- and sex-specific SDS of lean mass index of the child relative to the first quartile of energy-adjusted protein intake. Trend tests were conducted by using the quartiles of protein intake as a continuous variable in the model. ^1.^ *Model 1*: The vegetable and animal protein intake were additionally adjusted for each other. ^2.^ *Model 2*: *Model 1* further adjusted for maternal age, maternal educational level, maternal smoking and alcohol use during pregnancy, folic acid supplementation, maternal body mass index at enrolment, maternal energy and carbohydrate intake, gestational age at birth, gender of the child, breastfeeding practice 2 months postpartum, and screen time of the children at 6 years of age; ^3.^ Energy-adjusted protein intake using the nutritional residual method. **Abbreviations:** CI: confidence interval. SDS: standard deviation score.

**Supplemental Table 7.** Association between protein intake during pregnancy and childhood fat-free mass index at the age of 6 years in mothers without hypertensive complications in pregnancy, preterm child birth, or those who gave birth to a new-born with a birth weight outside ±2 SD (n=2,242)

|  | **Fat-free mass index** (SDS)  n=2,242 | |
| --- | --- | --- |
|  | ***Model 1*^1^** | ***Model 2*^2^** |
| **Total protein intake^3^** | β (95% CI) | β (95% CI) |
| Quartile 1 | *reference* | *reference* |
| Quartile 2 | **0.11 (0.06, 0.17)** | **0.11 (0.00, 0.22)** |
| Quartile 3 | **0.11 (0.05, 0.16)** | 0.09 (-0.02, 0.20) |
| Quartile 4 | **0.20 (0.14, 0.25)** | **0.18 (0.06, 0.29)** |
| *p for trend* | ***0.001*** | ***0.01*** |
| **Animal protein intake^3^** | |  |
| Quartile 1 | *reference* | *reference* |
| Quartile 2 | **0.08 (0.03, 0.13)** | 0.09 (-0.02, 0.20) |
| Quartile 3 | **0.11 (0.05, 0.17)** | 0.10 (-0.01, 0.21) |
| Quartile 4 | **0.17 (0.12, 0.23)** | **0.15 (0.03, 0.28)** |
| *p for trend* | ***0.002*** | ***0.02*** |
| **Vegetable protein intake^3^** | |  |
| Quartile 1 | *reference* | *reference* |
| Quartile 2 | **0.06 (0.01, 0.12)** | 0.08 (-0.03, 0.19) |
| Quartile 3 | **0.14 (0.09, 0.20)** | **0.16 (0.05, 0.27)** |
| Quartile 4 | **0.25 (0.19, 0.30)** | **0.25 (0.14, 0.37)** |
| *p for trend* | ***<0.001*** | ***<0.001*** |

Results from multivariable linear regression analyses, based on imputed data. The regression coefficients (95% CI) reflect the difference in age- and gender-specific SDS of the fat-free mass index of the child relative to the first quartile of energy-adjusted protein intake. Trend tests were conducted by using the quartiles of protein intake as a continuous variable in the model. ^1.^ *Model 1:* The vegetable and animal protein intake were additionally adjusted for each other. ^2.^ *Model 2*: *Model 1* further adjusted for maternal age, maternal educational level, maternal smoking and alcohol use during pregnancy, folic acid supplementation, maternal body mass index at enrolment, maternal energy and carbohydrate intake during pregnancy, gestational age at birth, gender of the child, breastfeeding practice 2 months postpartum, and screen time of the children at 6 years of age; ^3.^ Energy-adjusted protein intake using the nutritional residual method. **Abbreviations:** CI: confidence interval, SDS: standard deviation score.
